# In vitro evaluation of critical ultrafiltration fluxes and transmembrane pressure in a high flux dialyzer

**DOI:** 10.1038/s41598-025-08262-1

**Published:** 2025-07-05

**Authors:** Siavash Sohan Gir, Nathalie Gayrard, Alain Ficheux, Jonas Laget, Chantal Cazevieille, Àngel Argilés, Flore Duranton

**Affiliations:** 1 RD Néphrologie SAS, Montpellier, France; 2 BC2M, University of Montpellier, Montpellier, France; 3 INM, University of Montpellier, Montpellier, France; 4 Néphrologie Dialyse St Guilhem, Sète, France

**Keywords:** Haemodiafiltration, Ultrafiltration, Permeability, Membrane fouling, Critical flux, Haemodialysis, Fluid dynamics

## Abstract

In patients treated with haemodiafiltration, high convection volumes are considered beneficial. However it leads to pressure instability and membrane fouling. We aimed to identify critical ultrafiltration fluxes based on different approaches including the maximal global ultrafiltration coefficient (_G_K_D−UF_ max), and to test the influence of ultrafiltration on system stability and membrane fouling. Experiments of cross-flow filtration of a protein-containing fluid (cow milk) were performed. The ultrafiltration rate (Q_UF_) was sequentially modified using a peristaltic pump and transmembrane pressure (TMP) was recorded. _G_K_D−UF_ and TMP stability over time were assessed. Q_UF_ critical values were estimated from the _G_K_D−UF_, critical flux, irreversible fouling and sustainable flux approaches. Membrane fouling was observed by microscopy. Proteins from the feed, ultrafiltrate and retained on membrane were assessed by protein assays and SDS-PAGE. The _G_K_D−UF_ max approach identified Q_UF_ critical values close to the irreversible fouling and sustainable flux. When Q_UF_ exceeded critical values, major increase in TMP over time was observed and more clogged dialyzer fibres were detected. Utilizing Q_UF_ below the _G_K_D−UF_ max critical value lead to stable TMP over time and fewer clogged fibres therefore _G_K_D−UF_ max is helpful to identify the critical ultrafiltration rate and can be used to optimize ultrafiltration flow that prevent membrane fouling.

**Supplementary Information:**

The online version contains supplementary material available at 10.1038/s41598-025-08262-1.

## Introduction

Following earlier attempts^1^the first haemodialysis allowing a proper survival of a chronic renal failure patient was reported in 1960^2^. Blood filtration membranes have greatly evolved since then^3,4^ and new techniques incorporating convection, such as diafiltration^5^haemodiafiltration (HDF)^6^and on line HDF^7^have been developed to improve efficacy. Recent studies suggest that HDF with high convection volumes would improve patient survival^8,9^ and concerns regarding feasibility were raised^10^. The level of ultrafiltration rates (Q_UF_) required to achieve high convection volume HDF may result in high and unstable transmembrane pressure (TMP)^11^. This can trigger pre-set pressure alarms, treatment interruptions and reduce dialysis session efficacy. Identifying the optimal Q_UF_ expected to improve blood purification while preserving system stability is essential.

The ultrafiltration flux (Q_UF_) depends on hydrostatic pressure gradient (ΔP) dynamic viscosity (µ) and the sum of hydraulic resistances (ΣR), Eq. 1. The equation can be reformulated to include the membrane ultrafiltration coefficient *K*_*UF*_, which corresponds to membrane hydraulic permeability (Eqs. 1 and 2). For protein solutions, oncotic or osmotic pressure (Δπ) should also be considered (Eqs. 1 and 2).

1 $$K_{{UF}} ~ = ~\frac{{1~}}{{~\left( {\mu .~\Sigma R} \right)}}~ = ~\frac{{Q_{{UF}} ~}}{{~\Delta P~ - ~\Delta \pi }}~~~$$

2 $$Q_{{UF}} ~ = \frac{{\left( {\Delta P~ - ~\Delta \pi } \right)}}{{\left( {\mu .~\Sigma R} \right)}}~~ = ~K_{{UF}} ~.~\left( {\Delta P~ - ~\Delta \pi } \right)~~~~~$$

Solving Eq. 1 to identify the optimal Q_UF_ is challenging, notably because of changes in viscosity, osmotic pressure, and membrane fouling that occur both along the hollow-fibre membrane and over time^12^. However, the hydraulic permeability of the filtration system (_G_K_D−UF_), can easily be observed. _G_K_D−UF_ is defined as the ratio of the ultrafiltration rate (Q_UF_) to the transmembrane pressure (TMP), (Eq. 3).

3 $$_{{\text{G}}} {\text{K}}_{{{\text{D}} - {\text{UF}}}} = ~\frac{{Q_{UF}}}{{TMP}}$$

_G_K_D−UF_ differs from the dialyzer hydraulic permeability K_UF_^13^ and varies with the dialysis setting^14^. It can be seen as an efficacy parameter since a produced effect (Q_UF_) is divided by the effort needed (TMP). The _G_K_D−UF_ corresponds to the hydraulic permeability of the entire filtration system; it follows a concave parabolic function with increasing Q_UF_, which maximum (_G_K_D−UF _max) should correspond to the optimal Q_UF_^13,15^. We aimed to validate the _G_K_D−UF_ approach to identify optimal Q_UF_ in comparison with other techniques.

While dead-end filtration has a feed solution pushed through a filter that retains particles, haemodialysis is a tangential flow or cross-flow filtration, where the feed runs parallel to the membrane. Cross-flow filtration is used industrially with a variety of membranes and feeds for diverse applications such as concentration and purification. Cross-flow filtration minimizes membrane fouling, enhances fluxes and prolongs membrane life when maintained in proper condition. However, when ultrafiltration flow exceeds a critical value or “critical flux”^16,17^the system instability and undesired membrane fouling can be observed^18–20^.

Establishing optimal filtration conditions in haemodialysis and in industrial cross-flow systems share similarities. Determining and accounting for the critical ultrafiltration flux could improve filtration efficacy and stability over time. In the present study, we assessed operating conditions in an experimental cross-flow filtration system using milk as feed. We explored changes in TMP, protein removal and membrane fouling with different ultrafiltration rates and identified critical values for ultrafiltration according to the irreversible fouling, the sustainable flux and the _G_K_D−UF _max approaches.

## Results

### _G_K_D−UF_ max, irreversible fouling and maximum sustainable flux.

**Fig. 1:**
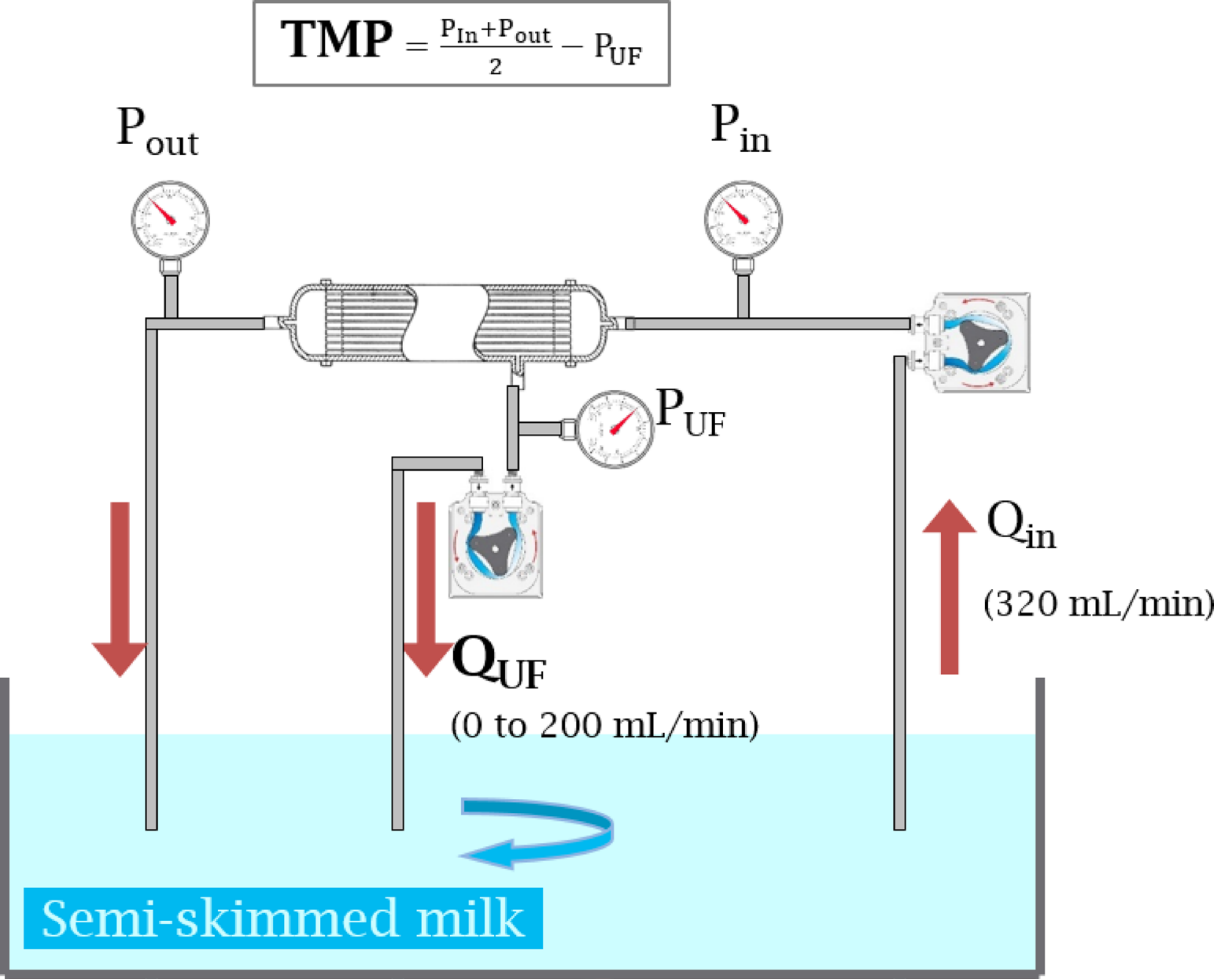
Diagram of the cross-flow filtration system used in all experiments.

Using a cross-flow setting with milk as feed solution (Fig. 1), we evaluated changes in TMP in response to different ultrafiltration fluxes, in order to identify the critical Q_UF_ flux corresponding to _G_K_D−UF_ max, irreversible fouling and maximum sustainable flux. Input flow was 318 ± 2 mL/min and a Gambro 210 H dialyzer was used (Table 1)

**Table 1:** Influence of ultrafiltration flux on transmembrane pressure and protein filtration.

Conditions	Condition 1		Condition 2		*P*-values
Time	T0	T60	T0	T60	
Q_in_ (mL/min)	322 ± 2		322 ± 2		0.9
Q_UF_ (mL/min)	126 ± 1		174 ± 4 ^†^		< 0.001
TMP (mmHg)	174 ± 4	179 ± 4	260 ± 15 ^†^	497 ± 20 ^†^*	< 0.001
Feed protein concentration (g/L)	31.6 ± 0.8	32.0 ± 1.2	30.1 ± 1.8	27.7 ± 2.9	0.4
Ultrafiltate protein concentration (mg/L)	36 ± 1	35 ± 1	52 ± 5 ^†^	38 ± 4 *	0.01
Sieving coefficient (‰)	1.14 ± 0.02	1.08 ± 0.06	1.72 ± 0.21 ^†^	1.35 ± 0.07	0.02
Proteins retained on the membrane (g)	-	0.91 ± 0.03	-	5.91 ± 0.30 ^†^	< 0.001

† Condition effect (*P* < 0.05 vs. condition 1 at same time).

* Time effect (*P* < 0.05 vs. T0 in same condition).

**Fig. 2:**
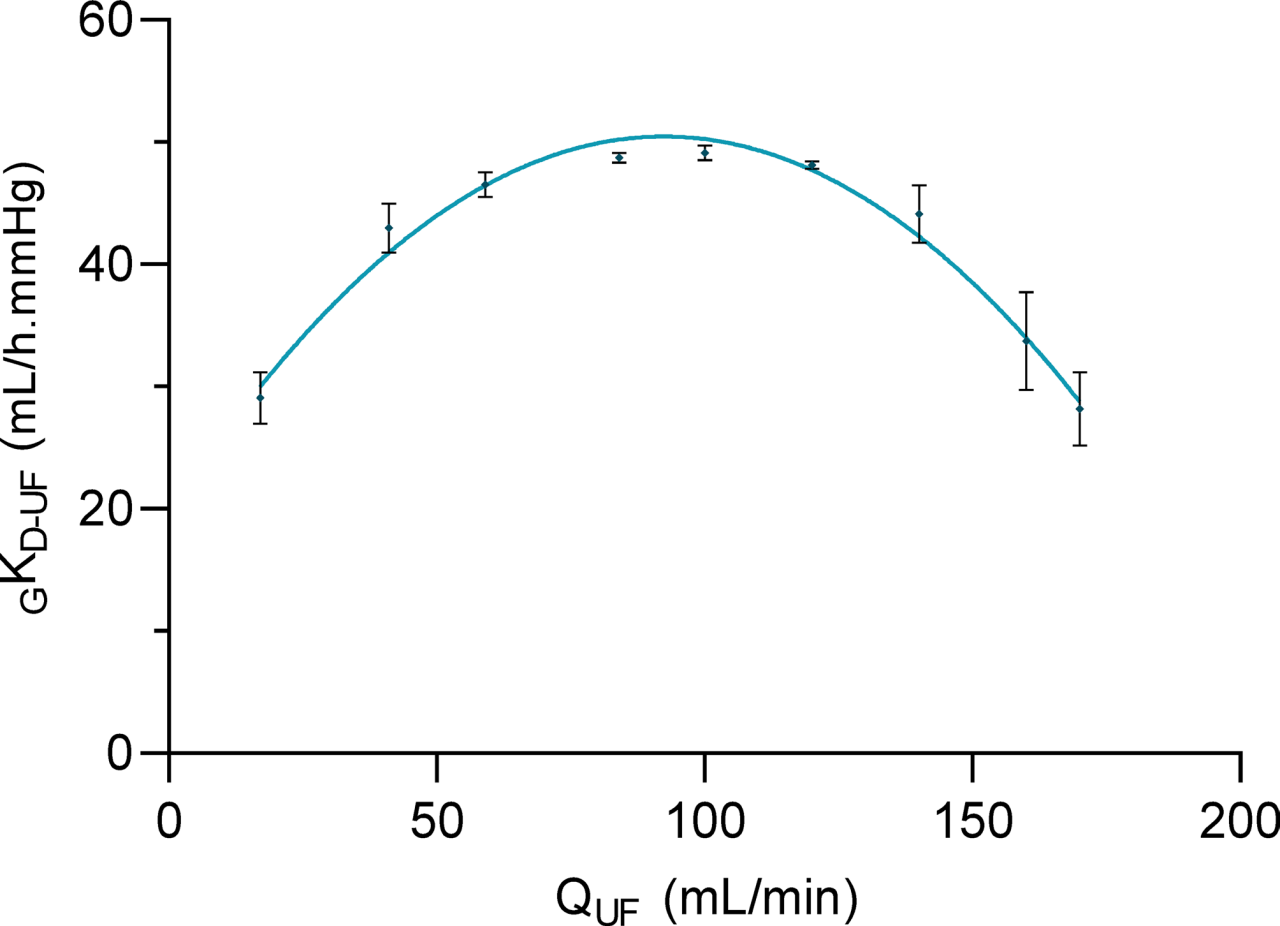
Global ultrafiltration coefficient (_G_K_D-UF_) observed at different ultrafiltration rates (Q_UF_). The maximum of the parabola corresponds to the critical ultrafiltration flux.

For the _G_K_D−UF_ max approach, Q_UF_ was increased in a stepwise manner. The global ultrafiltration coefficient _G_K_D−UF_ changed with ultrafiltration rate Q_UF_ (Fig. 2). It can be seen that the global ultrafiltration coefficient first increased and then dropped with increasing ultrafiltration rates. The parabolic regression nicely fits the observations (R² = 0.978). The mean Q_UF_ at _G_K_D−UF_ max was 95 ± 5 mL/min.

**Fig. 3:**
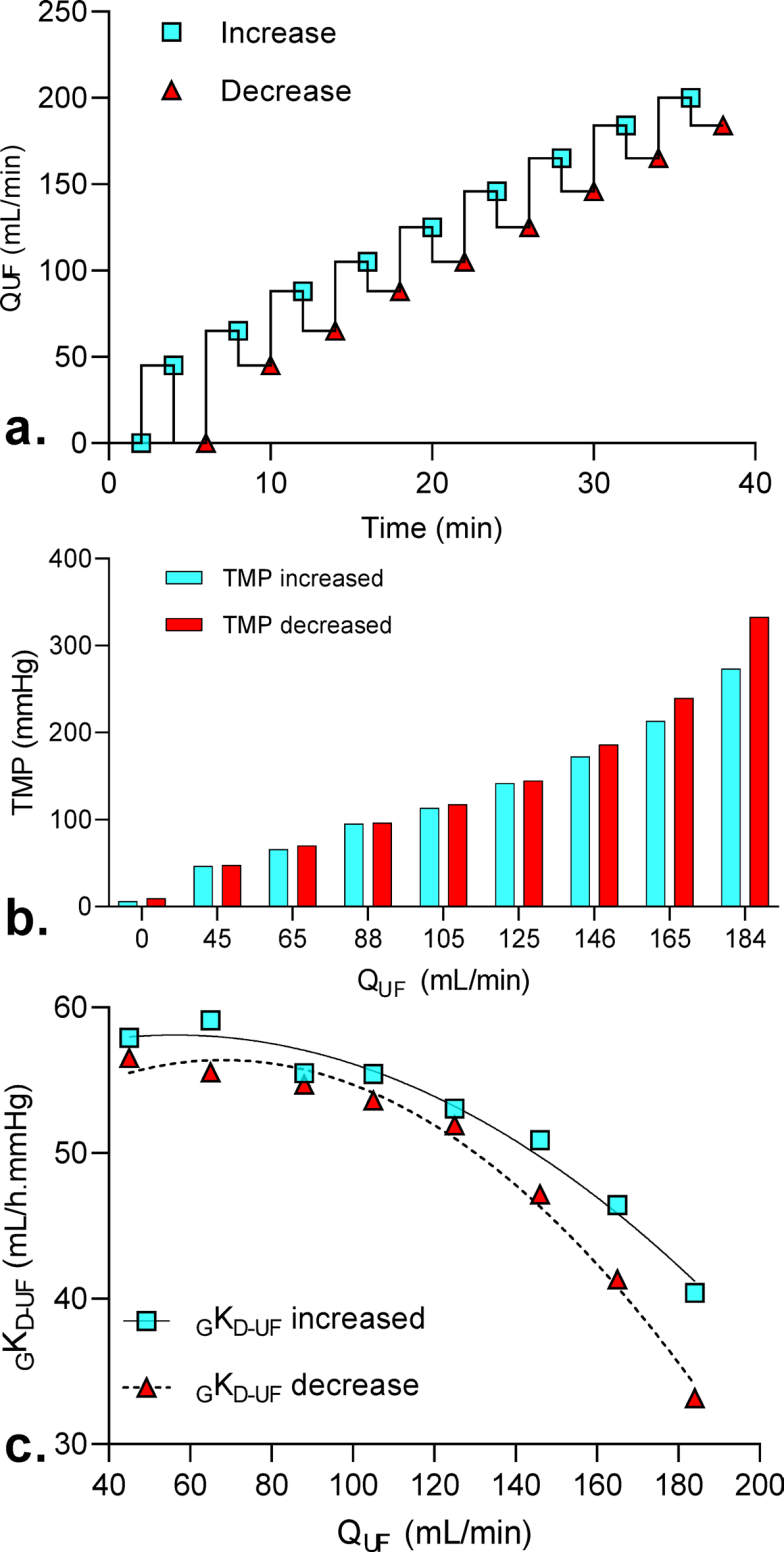
Influence of irreversible fouling on TMP and _G_K_D-UF_. (a) Successive increase and decrease in Q_UF_ were applied; (b) TMP was recorded at each Q_UF_ step and (c) _G_K_D-UF_ was calculated.

To detect the presence of irreversible membrane fouling, successive increases and decreases in ultrafiltration rate were applied (Fig. 3a), and TMP was recorded. We observed that TMP increases with Q_UF_. Interestingly, TMP was lower the first time it reached any given Q_UF_ value (in blue, Fig. 3b), than when the Q_UF_ was decreased to reach the same Q_UF_ value (in red). This was more obvious at higher Q_UF_ (above 125 mL/min), and was associated with a faster decline in _G_K_D−UF_ (Fig. 3c). This is a sign of irreversible membrane fouling during the short duration of the step, which by definition occurs when Q_UF_ exceeds the critical flux of the setting. The critical Q_UF_ determined by irreversible fouling was estimated at 115 ± 10 mL/min.

**Fig. 4:**
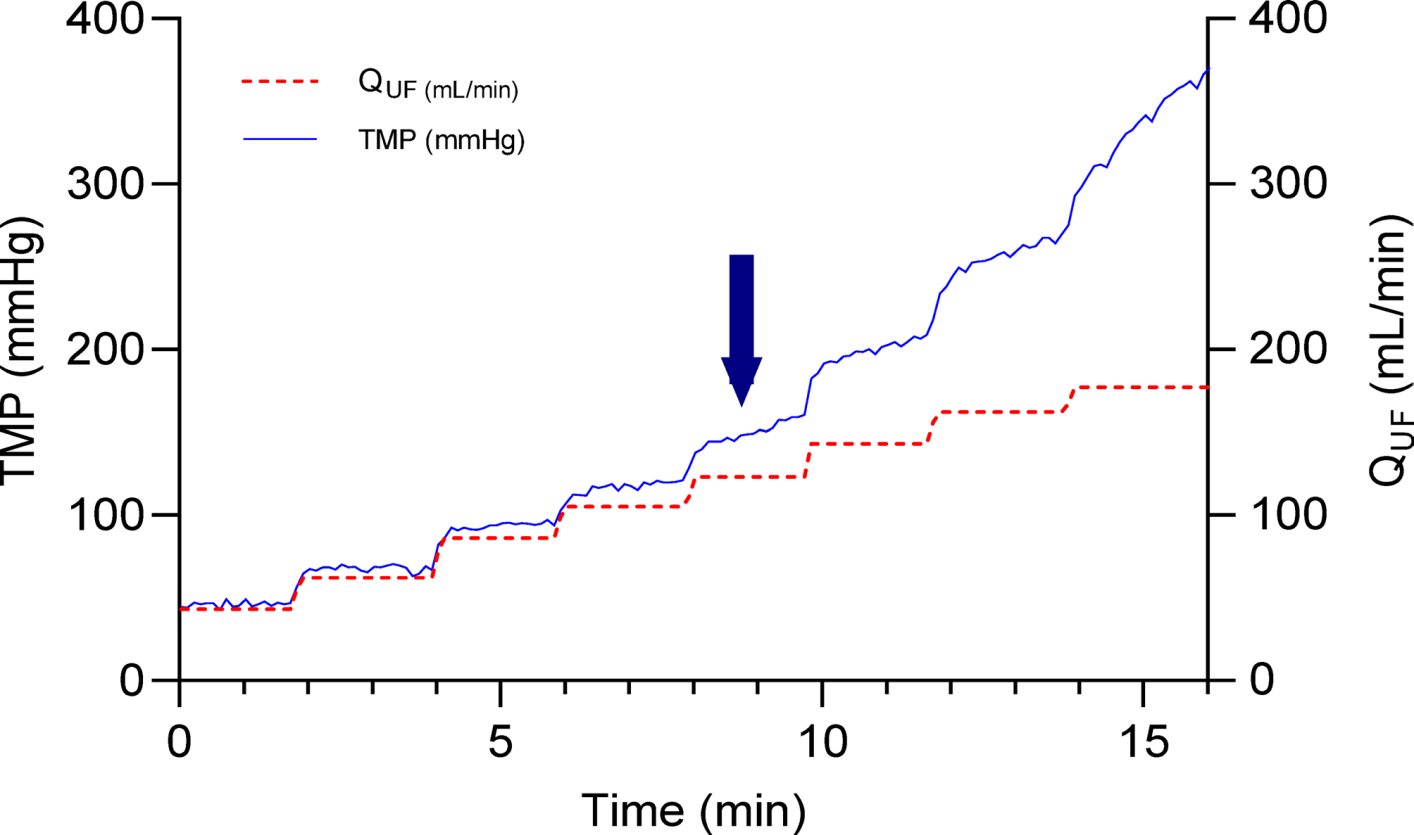
Investigation of sustainable flux by stepwise increase in Q_UF_ and TMP monitoring. The arrow shows Q_UF_ value when the TMP did not remain stable for a fixed Q_UF_.

Finally, to assess the maximum sustainable flux, Q_UF_ was again increased in a stepwise manner (Fig. 4). At Q_UF_ values below 120 mL/min, TMP was stable within each Q_UF_ step. Beyond this value (indicated by an arrow in Fig. 4), TMP increased while Q_UF_ was maintained stable. The critical flux defined as the maximum sustainable flux corresponds to the step directly preceding a clear increase in TMP. The critical Q_UF_ determined as maximum sustainable flux was estimated at 111 ± 6 mL/min.

Irreversible fouling and maximum sustainable flux were very close. The global ultrafiltration coefficient method identified a slightly lower to the other two methods.

### Critical flux, protein filtration and membrane fouling

To assess protein filtration and membrane fouling over time, we performed cross-flow filtration with different Q_UF_ flow rates but similar input flow rates (Table 2). We used FX100 dialyzers which led to slightly higher sustainable fluxes (126 ± 1 mL/min) compared to Gambro 210 H dialyzers (111 ± 6 mL/min). Cross-flow filtration was maintained 60 min in Condition 1 with the Q_UF_ at the value of sustainable flow or in Condition 2 where Q_UF_ exceeded the maximum sustainable flow (Table 2)^21^.

**Table 2:** Dialyser characteristics from manufacturers’ brochure.

Dialyzer (Commercial name)	210H^45^	FX-100^46^
Manufacturer	Gambro	Fresenius Medical Care AG & Co. KGaA
Material	Poliamix^®^	Helixone^®^
Composition	Polyarylethersulfone	polysulfone
Area (m^2^)	2.1	2.2
Wall thickness (µm)	50	35
Internal diameter (µm)	215	185
Membrane K_UF_(mL.h^−1^.mmHg^−1^)	85	73
Sieving coefficient	-	-
Inulin (5 kDa)	1.0	1.0
β2-microglobulin (11.8 kDa)	0.7	0.8
Albumin (65 kDa)	< 0.01	0.001

**Fig. 5:**
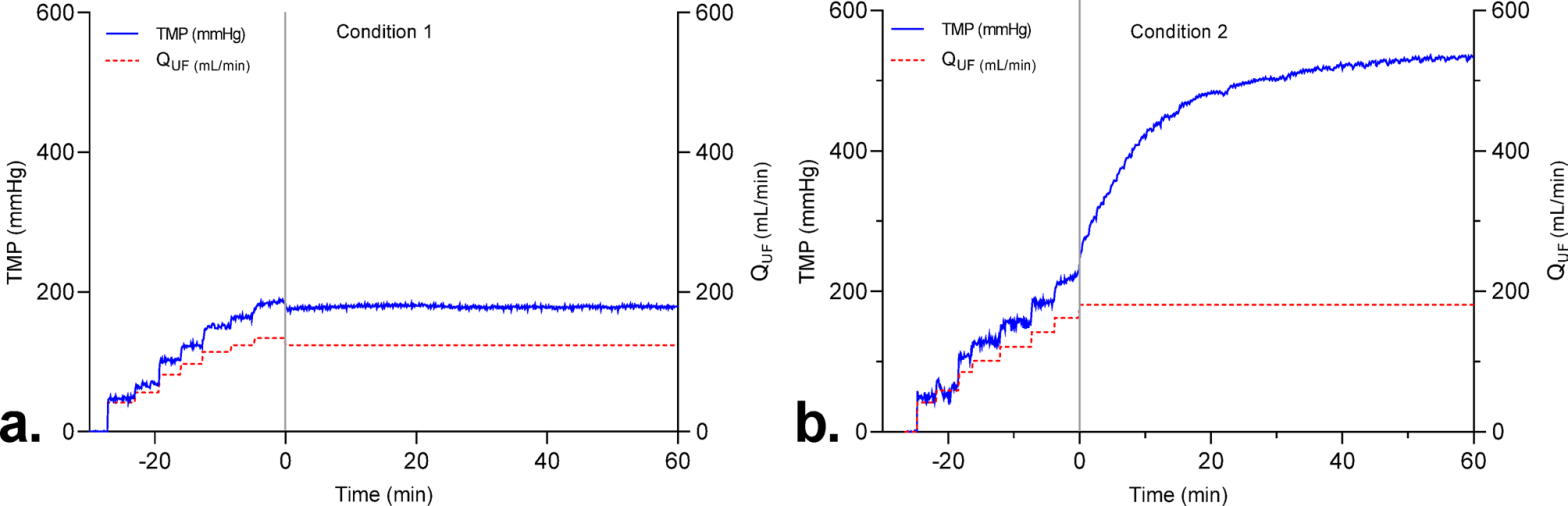
Influence of Q_UF_ settings on TMP over time. (a) In condition 1, sustainable flux value was identified and Q_UF_ was fixed at this value. (b) In condition 2, sustainable flux value was identified and Q_UF_ was fixed at 40% over this value.

At constant Q_UF_, it can be observed that TMP remained stable throughout the observation period in Condition 1 (Fig. 5a) while TMP increased in condition 2 and tended to plateau at a high value (Fig. 5b). The mean TMP values recorded at T_0_ andT_60_ (one hour later) confirmed that TMP remained stable and low in condition 1, while it was higher at T_0_ and largely increased at T_60_ in condition 2 (Table 2).

**Fig. 6:**
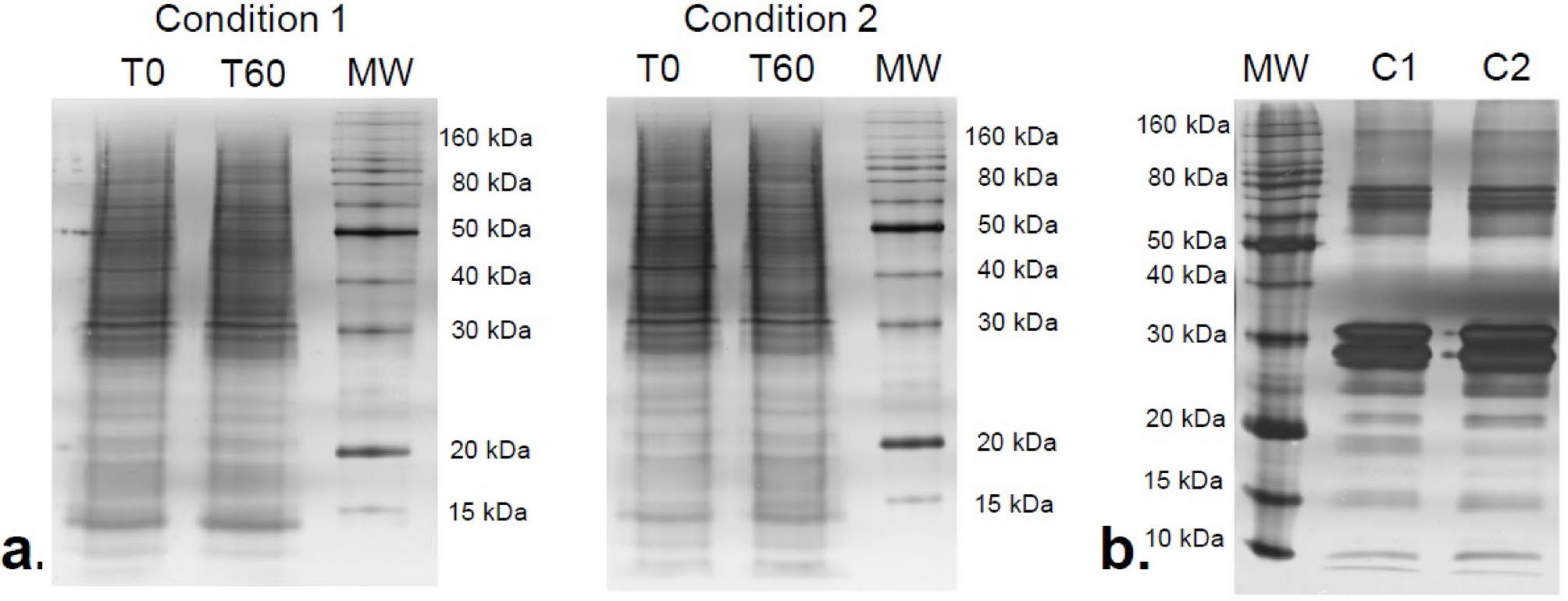
SDS-PAGE protein profiles of ultrafiltrate and proteins retained in the membrane. (a) SDS-PAGE protein profiles of ultrafiltrates are displayed for condition 1 and condition 2 at T0 and T60. (b) SDS-PAGE profiles of membrane retained proteins for condition 1 (C1) and condition 2 (C2).

At T_0_, total protein concentration in ultrafiltrate was higher in condition 2 than condition 1 (Table 2). It was also significantly decreased at T_60_ in condition 2, compared to T_0_. In contrast, total protein concentration in ultrafiltrate did not change over time in condition 1 (Table 2). To account for differences in protein concentration in the feed, sieving coefficients were calculated as the ratio of the concentrations of the ultrafiltrate to the feed. There was no change in protein sieving coefficient in condition 1 between T_0_ and T_60_ (Table 2). Again, the sieving coefficient was higher in condition 2 at T_0_ and decreased at T_60_ (Table 2). To assess if there were compositional changes in proteins in ultrafiltrate, we performed SDS-PAGE electrophoresis and found no changes in protein patterns in ultrafiltrate across the two conditions and time points (Fig. 6a). The uncropped gel blot image is available in the supplementary document (Fig. S1).

The total amount of proteins retained in the membrane in condition 2 was 6 times higher than in condition 1 (Table 2). The SDS-PAGE pattern of proteins retained in the membrane was similar across conditions, suggesting a quantitative change in protein adsorption rather than a qualitative change in this experiment (Fig. 6b). The uncropped gel blot image is available in the supplementary document (Fig. S2).

**Fig. 7:**
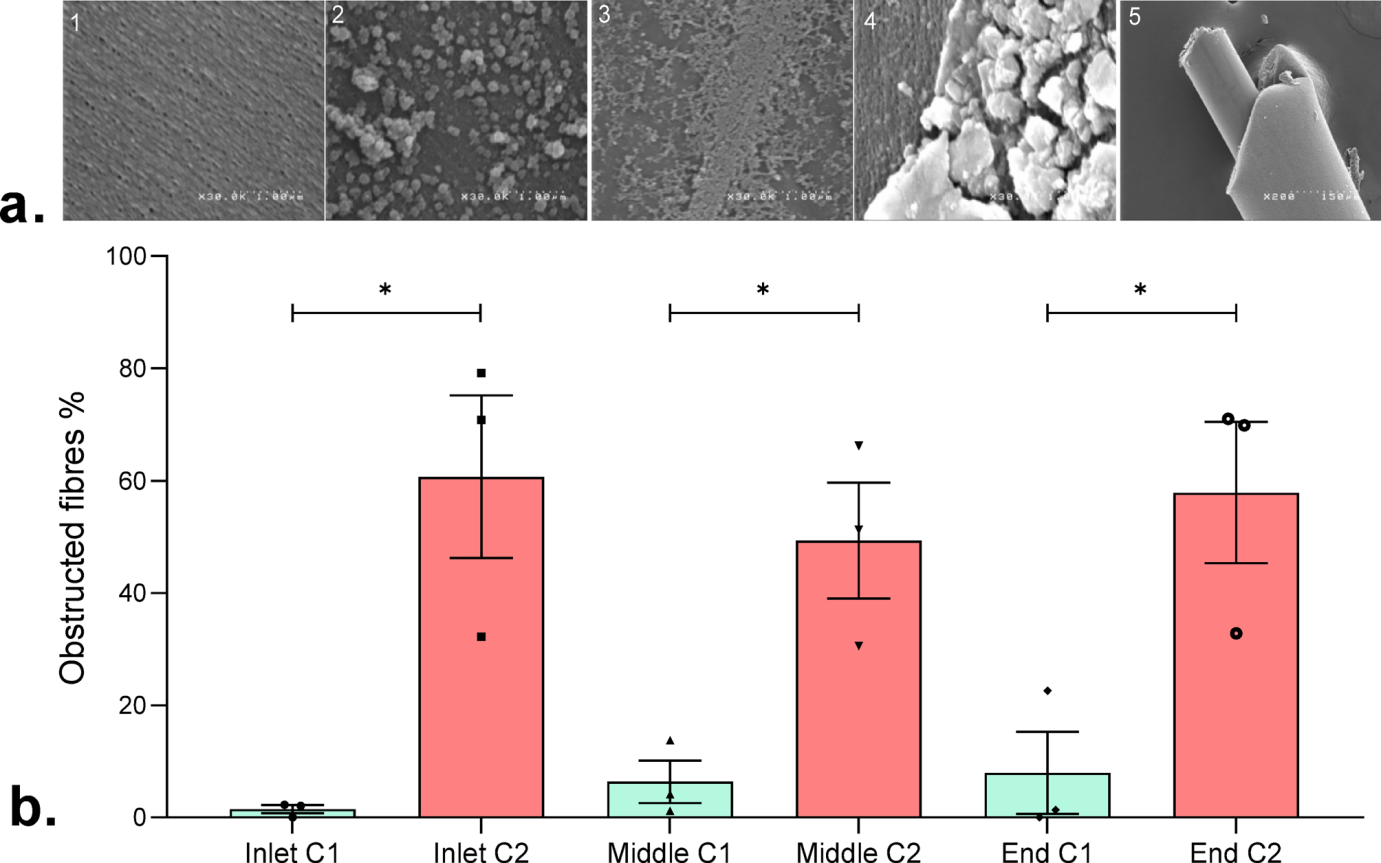
Investigation of membrane fouling. (a) Electron microscopy photographs of the inner side of membrane fibres (photographs 1-4, zoomed 30,000x) and a clogged fibre (image 5, zoomed 200x). Photograph 1 shows a clean fibre prior to a dialysis session. Photographs 2, 3 and 4 display material aggregation in the fibres. (b) The proportion of obstructed dialyser fibres found at the inlet, median and outlet of the dialyser in the 2 conditions. * indicates p < 0.05.

Finally, electron microscopy was performed to characterise the content of dialyzer fibres. A wide range of aggregated materials was observed in the membranes (Fig. 7a), going from no (image 1) or very few aggregates (images 2 and 3) to more abundant aggregates (image 4) and total obstruction of fibre lumens (image 5). Aggregates were observed in the two conditions. However, the proportion of fully obstructed fibres was significantly higher in condition 2, at any location within the dialyzer (inlet, centre or outlet). (Fig. 7b).

## Discussion

We have previously reported^13^ in extracorporeal blood filtration systems (haemodiafiltration) that _G_K_D−UF_ changes with ultrafiltration rate (Q_UF_) according _G_K_D−UF_ increases at low levels of Q_UF_, reaches a maximum value (vertex of the curve) and decreases thereafter with higher Q_UF_ levels^13,15^. We wanted to extend our observations to other cross-flow filtration systems and investigate the causes of membrane fouling and changes in efficacy of dialysis. In the present study, we observed that _G_K_D−UF_ also sharply decreased beyond a given value of Q_UF_ in a milk filtration system. Our studies show that the characteristic Q_UF_ that precedes the significant drop in _G_K_D−UF_, may be easily and quickly determined using a stepwise increase in ultrafiltration rate. This value is comparable to the values ​​determined by the two other methods of determining the critical flux. Protein removal by ultrafiltration, TMP and therefore _G_K_D−UF_ remained unchanged at critical Q_UF_, whilst irreversible membrane fouling occurred at higher Q_UF_ levels along with unstable and increasing TMP, and consequent drop in ultrafiltration coefficient. With regards to the stepwise approach, the parabolic regression of the global ultrafiltration coefficient is more flexible as the result is not strictly dependent of the selected Q_UF_ steps.

Changes in permeability over time of a cross-flow filtration system depend on factors linked to the membrane, the filtered fluid as well as factors modifying the applied pressure. Membrane-dependant factors influencing permeability over time include diameter and length of the membrane fibres, viscosity change and membrane fouling over time, as well as the initial pore diameter, pore number and distribution, membrane hydrophobicity and other factors^22^. The fluid-specific factors influencing membrane permeability are mainly dependant on viscosity and oncotic pressure^23^while the resulting pressure in the filtration system follows the Ernest Starling law (depending on hydrostatic and negative pressures applied to both sides of the membrane)^24^. It is noteworthy that some factors may influence both the fluid and the membrane, and may also modify the physics of the filtration system^25^. Typically, the formation of protein aggregates and membrane fouling is primarily dependent on the fluid constituents, but results in filtration membrane modifications and induces pressure changes in a constant ultrafiltration flow situation. It is also of interest that some factors may be modified by the filtration phenomenon and their modifications influence in turn, filtration yielding. For instance, Espinase et al.^26^evaluated oncotic pressure variations (Δπ) with ultrafiltered flow rate changes by square wave barovelocimetry and observed a 5-fold increase in Δπ in their setting with increasing Q_UF_ when it is known that Δπ in turn modulates ultrafiltration flow. Internal resistance related to flow and viscosity are also modified by Q_UF_^27^. Gradually adjusting the filtration flow rate can reduce the TMP and therefore the _G_K_D−UF_ can be higher than when the pressure is directly applied to the target value with a high constant flow^28^.

In our search for higher performance, we tend to operate the cross-flow filtration systems at high fluxes, increasing concentration polarization effects, which predispose to membrane fouling^17^. Chan et al.^29^using MALDI-MS quantitative analysis, identified variations in the protein layer along the fibres depending on flow, and colloidal surface interactions of proteins may play an important role in membrane fouling^30,31^. Our studies using two different flows showed that the amount of retained proteins and the number of clogged fibres increased significantly when the system was maintained with a high and constant Q_UF_ rate associated with a strong increase in TMP and therefore a decrease in _G_K_D−UF_ (condition 2). Therefore, using our system to establish the highest Q_UF_ level at which TMP remains relatively constant over time is useful in predicting or preventing the occurrence of protein aggregates and subsequent membrane fouling. Working at the Q_UF_ level that precedes a drop in the ultrafiltration coefficient is also relevant for monitoring protein transport, as the protein ultrafiltration flux and sieving coefficient were constant over time. Instead, when the cross-flow filtration system was subjected to the higher Q_UF_ level, the proteins sieving coefficient was initially higher, and it decreases over time (condition 2). This is consistent with colloid flux paradox described by Cohen et al.^32^: despite a lower diffusion coefficient of bigger particles, higher ultrafiltration rates increase their transport^33^. Although this suggests a shift towards bigger proteins in the ultrafiltrate in presence of higher sieving coefficient, this was not the observed in SDS-PAGE profiles.

Gésan et al.^34^using a microfiltration system maintained with a constant Q_UF_ for increasing time periods, demonstrated a differential fouling in the outlet as compared to the inlet or the middle part of the micro filters. They observed a higher percentage of fouling at the outlet than at the inlet by 60 min of microfiltration and this difference was blunted with time, as the percentage of fouling increased at the inlet whilst remained stable at the outlet areas. In our setting, using the higher Q_UF_ rate resulted in a high proportion of obstructed fibres at any place in the dialyzer, while the lower Q_UF_ rate condition prevented fibre obstruction. However, there was no evidence of a differential fouling of fibres along the dialyser after 60 min of ultrafiltration.

The advantages of seeking a relatively stable flow over time in cross-flow filtration systems to protect the membrane from detrimental clogging are obvious. The threshold separating the filtration flow with beneficial effects from that with detrimental consequences on the filtration system may vary depending on the applications and needs to be determined based upon the critical or sustainable flux. For milk filtration it has been previously shown that the flux at which membrane fouling becomes irreversible is close to the flux at which ultrafiltration is no longer sustainable^35^. The methods to calculate a critical flux require determining all the factors influencing the cross–filtration system, rendering its determination cumbersome, submitted to cumulative error factors and uneasy to be applied to any automatic tool designed to control filtration yielding and stability^36^. This is particularly true for protein containing solutions or macromolecules with different physico-chemical properties that may form agglomerates in the membrane or interact with each other and change their diffusive properties or their osmotic pressures^37,38^.

Our present study demonstrates that assessing ultrafiltration rate and pressure at the outlet and inlet of the system allows determining ultrafiltration coefficient and identifying the Q_UF_ rate at which _G_K_D−UF_ drop occurs. Permeability determination of a cross–filtration system bypasses most of the methodology associated problems as it determines the global performance of the system and is easily obtained.

The critical ultrafiltration flux assessment by the _G_K_D−UF_ max method of a membrane filtration system is a simple and rapid method to find the optimal ultrafiltration flow that prevents membrane fouling. This critical value is close to those found with other methods used in industry. The clinical relevance of accounting for the critical flux in HDF should be further investigated.

## Methods

### Experimental cross-filtration setting

 To simulate HDF in vitro, we established a cross-flow filtration system (Fig. 1) using peristaltic pumps of a haemodialysis generator (Gambro AK200, Lundia AB, Lund, Sweden), and a high permeability hollow fibre dialyser as filter. Tests were performed with two dialyzers (Table 1) to improve generalizability. Three litres of semi-skimmed UHT cow’s milk were used for each experiment. Milk was selected for its colloidal nature^39^ and protein content (33 g/L)^40^, expected to reproduce membrane fouling observed in HDF. Semi-skimmed UHT milk also contains carbohydrates (48 g/L) and fat (16 g/L)^41^. Using the Bradford method, we found an average protein concentration of 31.7 ± 0.7 g/L in milk at the start of the experiments.

A peristaltic pump generated a constant inlet flow rate of feed solution from reservoir to the dialyser inlet, Q_in_, set at 320 mL/min. The second pump controlled the ultrafiltration rate (Q_UF_) from the ultrafiltrate outlet and back into the reservoir. Flows were recorded and flow rate accuracy was checked by collecting and weighing the feed and ultrafiltrate output over a given period of time. The pressures at feed inlet (P_in_), feed outlet (P_out_), and ultrafiltrate outlet (P_UF_) were recorded using pressure gauges (HDM97, IBP Instruments GmbH, Hannover, Germany). Transmembrane pressure (TMP) was calculated according to Eq. 4.

4 $${\text{TMP}} = \frac{{P_{{In}} + P_{{out}} }}{2} - P_{{UF}}$$

Several cross-filtration experiments were performed. Milk and dialyzers were renewed for each experiment. All measurements were performed at room temperature (22°C).

## Determining critical ultrafiltration flux

*Global ultrafiltration coefficient*: The ultrafiltration rate was increased in a stepwise manner from 20 to 170 mL/min, by 20 to 30 mL/min steps, and maintained for 2 min. TMP was recorded after stabilization or, when unstable, after 2 min. The global ultrafiltration coefficient _G_K_D−UF_ was calculated from Eq. 3.

*Irreversible fouling : *Based on the work by Wu et al.^42^ and Espinase et al.^27^the ultrafiltration rate was changed every 2 min by increasing steps of 40 mL/min, followed by decreasing steps of 20 mL/min, and TMP was recorded. The critical flux was identified as the ultrafiltration rate beyond which TMP increases while maintained in constant ultrafiltration rate, signifying that irreversible membrane fouling occurred.

*Sustainable flux:* Flux was increased in a stepwise manner from 45 mL/min to 200 mL/min. Each ultrafiltration rate was maintained for 2 min and TMP was recorded. The maximum sustainable flux was identified as the ultrafiltration rate preceding a clear increase in TMP.

### Cross-filtration experiment

*Conditions:*We determined critical ultrafiltration rate by sustainable flux method at the start and performed in vitro cross-filtration for at least 60 min, setting Q_UF_ at the sustainable flux (condition 1) or 40% higher (condition 2). Pressures were recorded during the experiment.

*Samples:* Feed and ultrafiltrate were sampled at the beginning of Q_UF_ stabilisation (T_0_) and after 1 h (T_60_) of cross-flow filtration. At the end of the experiment, dialysers were rinsed with 2 L of saline solution. After draining, dialysers were refilled with 200 mL of 3 mM EDTA/PBS 1X and the solution was recirculated for 30 min at 80mL/min at room temperature and sampled. Dialyser shells were then cut with a saw and fibres were recovered. A sample of fibres was taken at the inlet at the centre and at the outlet of the dialyser for microscopy studies. Then proteins were extracted from the membranes by soaking in 1% SDS and sonication for 5 min at room temperature. Fibres were removed from the solution and protein assays were performed. By the concentrations and volumes, the total mass of proteins extracted from the fibres was calculated.

*Protein assays:* The total protein concentration in the ultrafiltrate at T_0_ and T_60_ and the amount of proteins retained on the membrane were determined using the Bradford method adapted for the low concentration range as previously described^43^ and by a BCA protein assay kit (Thermoscientific, Il, USA). SDS-PAGE was performed according to the method described by Laemmli^44^ using a Bio-Rad system (Bio-Rad laboratories, CA, USA). Approximately 1 µg of protein in 2% SDS sample buffer was run in a 12.5% acrylamide gel and then stained with a silver-stained kit (Invitrogen, CA, USA). SDS-PAGE gels were scanned with an Epson Perfection 4990 PHOTO (Epson, CA, USA).

*Electron microscopy:* Fibres were fixed in 2.5% glutaraldehyde overnight at 4 °C and the next day progressively dehydrated using a graded (30 to 100%) ethanol series. Then fibres were treated with hexamethyldisilazane for 90 s, dried, cut with a scalpel under a binocular microscope to see inside the fibres and to count those that were clogged. Fibres were coated with gold-palladium, and examined under a scanning electron microscope (Hitachi 4000 at INM Montpellier, France).

### Statistical analysis

Results are presented as timed series from individual measurement series or as mean and standard error of experimental replicates. Differences between groups was assessed by Two-Tailed unpaired t-test, and Analysis of variance (ANOVA) followed by Tukey’s tests using GraphPad Prism version 9.0.0 (Boston, USA). All results are presented as mean ± standard error of the mean.

## Electronic supplementary material

Below is the link to the electronic supplementary material.

Supplementary Material 1

## Data Availability

The datasets generated and analysed during the current study are available from the corresponding author upon request.
