# Supplementary material for: In vitro evaluation of critical ultrafiltration fluxes and transmembrane pressure in a high flux dialyzer
Source: Sci Rep. 2025 Jul 5;15:24083. doi: 10.1038/s41598-025-08262-1 (PMC12228744; doi:10.1038/s41598-025-08262-1)

In vitro evaluation of critical ultrafiltration fluxes and transmembrane pressure in a high flux dialyzer

Siavash SOHAN GIR, Nathalie GAYRARD, Alain FICHEUX, Jonas LAGET, Chantal CAZEVIEILLE, Àngel ARGILÉS, Flore DURANTON

Supplementary Information

This supplement contains raw images of the SDS-PAGE protein profiles presented in figure 6 of the original article.

Figure S1 Raw gel blots of figure 6a, SDS-PAGE protein profiles of ultrafiltrates for condition 1 and condition 2 at T0 and T60 with molecular weight markers (WM).

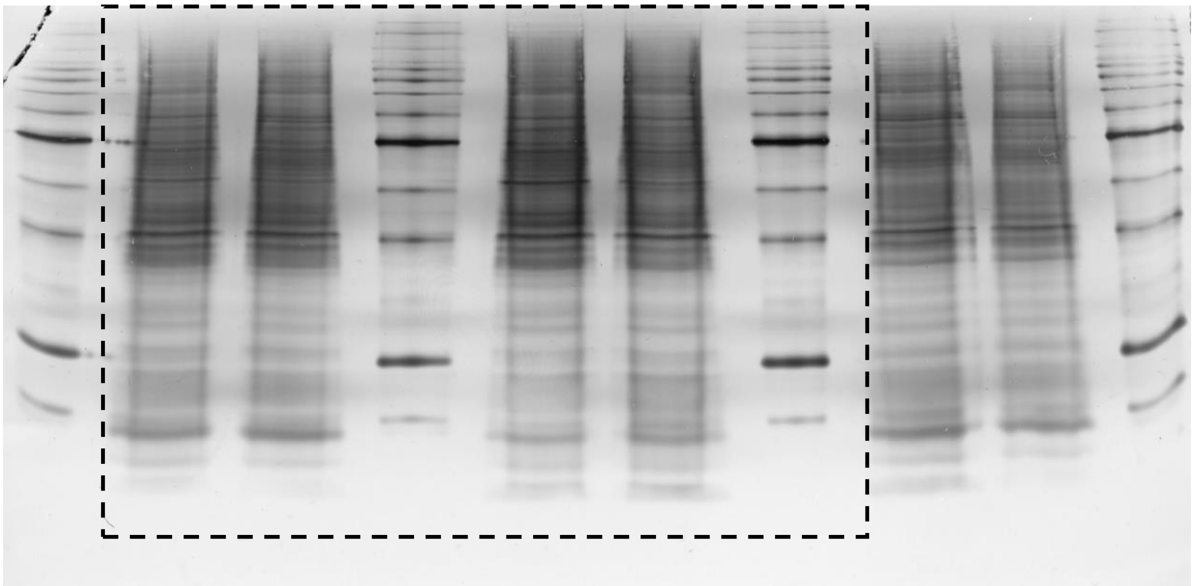

Figure S2 Raw gel blots of figure 6b, SDS-PAGE profiles of membrane retained proteins for condition 1 and condition 2 with molecular weight markers.

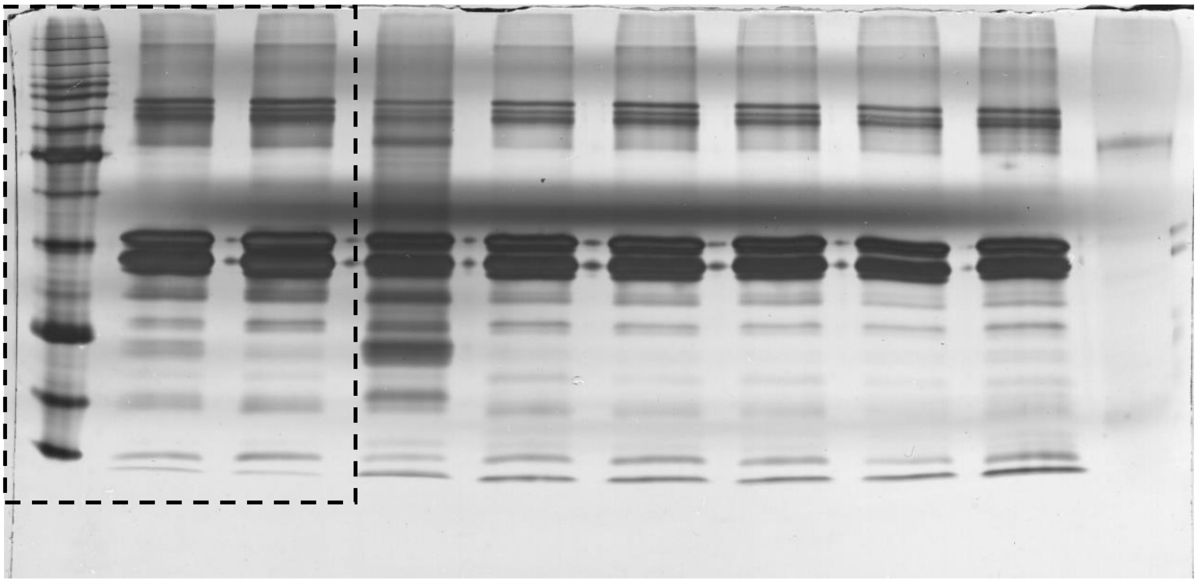

Supplement: Supplementary file 1 — Supplementary Material 1 [file 41598_2025_8262_MOESM1_ESM.pdf]
